# Supplementary material for: Genetic polymorphisms of superoxide dismutase 1 are associated with the serum lipid profiles of Han Chinese adults in a sexually dimorphic manner
Source: PLoS One. 2020 Jun 19;15(6):e0234716. doi: 10.1371/journal.pone.0234716 (PMC7304602; doi:10.1371/journal.pone.0234716)
Supplement: S1 Fig — (A) Results of Tagger. (B) Linkage disequilibrium plot showing r2 multiplying 100. (DOCX) [file pone.0234716.s001.docx]

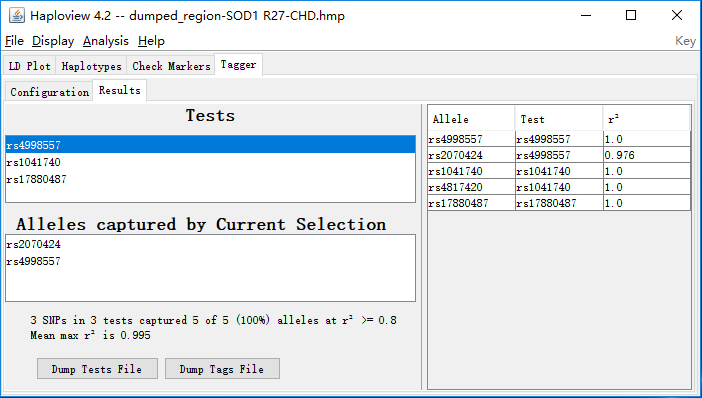


S1 Fig (A)


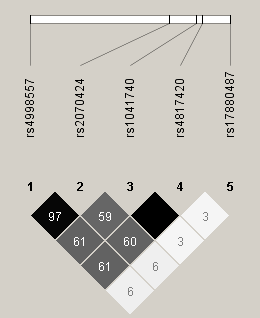


S1 Fig (B)

**S1 Fig.** **Selection of tag single nucleotide polymorphisms of superoxide dismutase 1 with Haploview software.** (A) Results of Tagger. (B) Linkage disequilibrium plot showing *r*^2^ multiplying 100.
