# Supplementary material for: Genetic polymorphisms of superoxide dismutase 1 are associated with the serum lipid profiles of Han Chinese adults in a sexually dimorphic manner
Source: PLoS One. 2020 Jun 19;15(6):e0234716. doi: 10.1371/journal.pone.0234716 (PMC7304602; doi:10.1371/journal.pone.0234716)
Supplement: S4 Fig — Genotyping the single nucleotide polymorphism of rs17880487 in superoxide dismutase 1 gene by melting curve analysis in molecular beacon probe-based qPCR experiment (A) and confirmation with Sanger sequencing (B). (A) Melting curve diagram generated by the original data collected with Roche 480 II qPCR instrument. (B) Sequencing analysis of the corresponding qPCR products with ▲ indicating the allelic loci. (DOCX) [file pone.0234716.s004.docx]

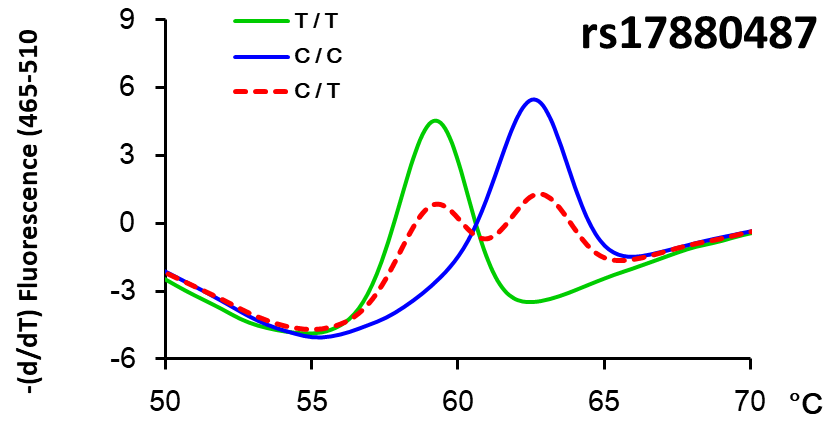
 S4 Fig (A)

 S4 Fig (B)

**S4 Fig.** **Genotyping the single nucleotide polymorphism of rs17880487 in superoxide dismutase 1 gene by melting curve analysis in molecular beacon probe-based qPCR experiment (A) and confirmation with Sanger sequencing (B).** (A) Melting curve diagram generated by the original data collected with Roche 480 II qPCR instrument. (B) Sequencing analysis of the corresponding qPCR products with ▲ indicating the allelic loci.
