# Supplementary material for: Genetic polymorphisms of superoxide dismutase 1 are associated with the serum lipid profiles of Han Chinese adults in a sexually dimorphic manner
Source: PLoS One. 2020 Jun 19;15(6):e0234716. doi: 10.1371/journal.pone.0234716 (PMC7304602; doi:10.1371/journal.pone.0234716)
Supplement: S1 Table — a Abbreviations: 3’UTR, untranslated region; BP, backward primer; FP, forward primer; MAF, minor allele frequency; Pr, Probe; SNPs, single nucleotide polymorphisms. b The global MAF values were cited from 1000Genomes as indicated in the NCBI SNP database. c The bases in small letters is the adapter sequence to form the stem of the beacon probe. The base in bold underlined italics is one of the allele for the SNP. d “Yes” indicated the primer used for Sanger sequencing of the amplicon to confirm the genotypes judged by the melting curves in qPCR experiment. (DOCX) [file pone.0234716.s005.docx]

**S1 Table. Primers and molecular beacon probes to genotype the tag SNPs of human superoxide dismutase 1 gene** ^a^

| Tag SNPs | Allele | Global MAF ^b^ | Location | Oligos, 5’-3’ ^c^ | Sequencing primer ^d^ | Amplicon length |
| --- | --- | --- | --- | --- | --- | --- |
| rs4998557 | G>A | 0.3291 | 1^st^ intron | FP: TTGATTTCTGTATGTAGCCACGGAGCA | Yes | 170 bp |
|  |  |  |  | BP: CAGGAGAGGACTGATTCTAGTCTA |  |  |
|  |  |  |  | Pr: cgtaaCCTGAATGGCTATACTGCTT***A***Cg |  |  |
| rs1041740 | C>T | 0.2428 | 4^th^ intron | FP: GACTTGTTTAACTTGTGGGAAGCTGTT | Yes | 137 bp |
|  |  |  |  | BP: GAATGGAAGTGACTACTGAATGTTA |  |  |
|  |  |  |  | Pr: cgccGCAAAA***C***ACCAAGTAGACAGGC |  |  |
| rs17880487 | C>T | 0.0142 | 5^th^ exon, 3’UTR | FP: GTGAATAAAAACCCTGTATGGCA |  | 128 bp |
|  |  |  |  | BP: CACTGTTAGTATTACTAAATCTGTTCCACTGAAGC | Yes |  |
|  |  |  |  | Pr: ccgagGTTTGAATTT***G***GATTCTTTTAATAGCCTCgg |  |  |

^a^ Abbreviations: 3’UTR, untranslated region; BP, backward primer; FP, forward primer; MAF, minor allele frequency; Pr, Probe; SNPs, single nucleotide polymorphisms.

^b^ The global MAF values were cited from 1000Genomes as indicated in the NCBI SNP database.

^c^ The bases in small letters is the adapter sequence to form the stem of the beacon probe. The base in bold underlined italics is one of the allele for the SNP.

^d^ “Yes” indicated the primer used for Sanger sequencing of the amplicon to confirm the genotypes judged by the melting curves in qPCR experiment.
