# Supplementary material for: Genetic polymorphisms of superoxide dismutase 1 are associated with the serum lipid profiles of Han Chinese adults in a sexually dimorphic manner
Source: PLoS One. 2020 Jun 19;15(6):e0234716. doi: 10.1371/journal.pone.0234716 (PMC7304602; doi:10.1371/journal.pone.0234716)
Supplement: S2 Table — a Abbreviations: HDLC, high-density lipoprotein cholesterol; LDLC, low-density lipoprotein cholesterol; TC, total cholesterol; TG, triglyceride. b Including those meeting the diagnosis criteria recommended by the Chinese guideline for dyslipidemia management* and those previously diagnosed as dyslipidemia patients. The prevalence of dyslipidemia between males and females was significantly different, P < 0.001. *Reference information: Joint committee for guideline revision. 2016 Chinese guidelines for the management of dyslipidemia in adults. J Geriatr Cardiol. 2018;15(1):1–29. Epub 2018/02/13. doi: 10.11909/j.issn.1671-5411.2018.01.011. PubMed PMID: 29434622; PubMed Central PMCID: PMCPMC5803534. (DOCX) [file pone.0234716.s006.docx]

**S2 Table.** **Composition of dyslipidemia in male and female subjects** ^a^

| Dyslipidemia criteria ^b^ | Male, *n* =1110 | |  | Female, *n* =1511 | |  | Total, *n* = 2621 | |
| --- | --- | --- | --- | --- | --- | --- | --- | --- |
|  | *n* | % |  | *n* | % |  | *n* | % |
| TG ≥ 2.3, mmol/L | 269 | 24.2 |  | 115 | 7.6 |  | 384 | 14.7 |
| TC ≥ 6.2, mmol/L | 129 | 11.6 |  | 189 | 12.5 |  | 318 | 12.1 |
| LDLC ≥ 4.1, mmol/L | 20 | 1.8 |  | 41 | 2.7 |  | 61 | 2.3 |
| HDLC < 1.0, mmol/L | 28 | 2.5 |  | 34 | 2.3 |  | 62 | 2.4 |
| Previously diagnosed dyslipidemia | 138 | 12.4 |  | 95 | 6.3 |  | 233 | 8.9 |
| Dyslipidemia ^b^ | 370 | 33.3 |  | 303 | 20.1 |  | 673 | 25.7 |

^a^ Abbreviations: HDLC, high-density lipoprotein cholesterol; LDLC, low-density lipoprotein cholesterol; TC, total cholesterol; TG, triglyceride.

^b^ Including those meeting the diagnosis criteria recommended by the Chinese guideline for dyslipidemia management* and those previously diagnosed as dyslipidemia patients. The prevalence of dyslipidemia between males and females was significantly different, *P* < 0.001.

* Reference information: Joint committee for guideline revision. 2016 Chinese guidelines for the management of dyslipidemia in adults. J Geriatr Cardiol. 2018;15(1):1-29. Epub 2018/02/13. doi: 10.11909/j.issn.1671-5411.2018.01.011. PubMed PMID: 29434622; PubMed Central PMCID: PMCPMC5803534.
