# Supplementary material for: Genetic polymorphisms of superoxide dismutase 1 are associated with the serum lipid profiles of Han Chinese adults in a sexually dimorphic manner
Source: PLoS One. 2020 Jun 19;15(6):e0234716. doi: 10.1371/journal.pone.0234716 (PMC7304602; doi:10.1371/journal.pone.0234716)
Supplement: S3 Table — a Abbreviation: SNPs, single nucleotide polymorphisms. (DOCX) [file pone.0234716.s007.docx]

**S3 Table. Comparisons of the genotype frequencies of three tag SNPs of superoxide dismutase 1 gene between adult males and females** ^a^

| Polymorphism ID | Genotype | Male, % | Female, % | *χ^2^* | *P*-value |
| --- | --- | --- | --- | --- | --- |
| rs4998557 | GG | 25.5 | 27.3 | 1.545 | 0.462 |
|  | AG | 48.9 | 46.7 |  |  |
|  | AA | 25.6 | 26.0 |  |  |
|  | G | 50.0 | 50.7 | 0.256 | 0.613 |
|  | A | 50.0 | 49.3 |  |  |
| rs1041740 | CC | 42.8 | 43.5 | 2.293 | 0.318 |
|  | CT | 45.8 | 43.4 |  |  |
|  | TT | 11.4 | 13.1 |  |  |
|  | C | 65.7 | 65.2 | 0.134 | 0.714 |
|  | T | 34.3 | 34.8 |  |  |
| rs17880487 | CC | 89.7 | 89.0 | 0.432 | 0.806 |
|  | CT | 9.9 | 10.5 |  |  |
|  | TT | 0.4 | 0.5 |  |  |
|  | C | 94.7 | 94.3 | 0.409 | 0.522 |
|  | T | 5.3 | 5.7 |  |  |

^a^ Abbreviation: SNPs, single nucleotide polymorphisms.
