# Supplementary material for: Genetic polymorphisms of superoxide dismutase 1 are associated with the serum lipid profiles of Han Chinese adults in a sexually dimorphic manner
Source: PLoS One. 2020 Jun 19;15(6):e0234716. doi: 10.1371/journal.pone.0234716 (PMC7304602; doi:10.1371/journal.pone.0234716)
Supplement: S4 Table — a Abbreviations: HDLC, high-density lipoprotein cholesterol; LDLC, low-density lipoprotein cholesterol; SNPs, single nucleotide polymorphisms; TC, total cholesterol; TG, triglyceride. b Multiple comparisons revealed the significant difference of genotype frequencies of GA and AA between low and normal HDLC groups, P < 0.05. c Genotype frequencies of TT was too low and did not meet the criteria for Chi-square test, so genotype CT and TT were combined. (DOCX) [file pone.0234716.s008.docx]

**S4 Table. Genotype frequencies of three tag SNPs of superoxide dismutase 1 gene in abnormal and normal lipid groups of adult males** ^a^

|  |  | TG | |  |  | TC | |  |  | LDLC | |  |  | HDLC | |  |  |
| --- | --- | --- | --- | --- | --- | --- | --- | --- | --- | --- | --- | --- | --- | --- | --- | --- | --- |
| mmol/L | | < 1.7 | ≥ 1.7 |  |  | < 5.2 | ≥ 5.2 |  |  | < 3.4 | ≥ 3.4 |  |  | ≥ 1.0 | < 1.0 |  |  |
| *n* | | 675 | 435 |  |  | 604 | 506 |  |  | 897 | 213 |  |  | 1082 | 28 |  |  |
| Genotype | | % | | *χ^2^* | *P* | % | | *χ^2^* | *P* | % | | *χ^2^* | *P* | % | | *χ^2^* | *P* |
| rs4998557 | GG | 25.6 | 25.3 | 0.02 | 0.99 | 24.5 | 26.7 | 0.94 | 0.63 | 25.1 | 27.2 | 1.04 | 0.60 | 25.5 | 25.0 | 7.31 | **0.03** ^b^ |
|  | GA | 48.9 | 49.0 |  |  | 49.0 | 48.8 |  |  | 48.7 | 49.8 |  |  | 49.4 | 28.6 |  |  |
|  | AA | 25.5 | 25.7 |  |  | 26.5 | 24.5 |  |  | 26.2 | 23.0 |  |  | 25.0 | 46.4 |  |  |
|  | G | 50.1 | 49.8 | 0.02 | 0.89 | 49.0 | 51.1 | 0.95 | 0.33 | 49.4 | 52.1 | 0.98 | 0.32 | 50.2 | 39.3 | 2.62 | 0.11 |
|  | A | 49.9 | 50.2 |  |  | 51.0 | 48.9 |  |  | 50.6 | 47.9 |  |  | 49.8 | 60.7 |  |  |
| rs1041740 | CC | 42.7 | 43.0 | 0.49 | 0.78 | 41.2 | 44.7 | 2.76 | 0.25 | 42.7 | 43.2 | 2.95 | 0.23 | 43.0 | 35.7 | 1.37 | 0.50 |
|  | CT | 46.4 | 44.8 |  |  | 48.0 | 43.1 |  |  | 46.6 | 42.3 |  |  | 45.7 | 46.4 |  |  |
|  | TT | 11.0 | 12.2 |  |  | 10.8 | 12.3 |  |  | 10.7 | 14.6 |  |  | 11.3 | 17.9 |  |  |
|  | C | 65.9 | 65.4 | 0.05 | 0.83 | 65.2 | 66.2 | 0.23 | 0.63 | 66.0 | 64.3 | 0.43 | 0.51 | 65.9 | 58.9 | 1.16 | 0.28 |
|  | T | 34.1 | 34.6 |  |  | 34.8 | 33.8 |  |  | 34.0 | 35.7 |  |  | 34.1 | 41.1 |  |  |
| rs17880487 | CC | 89.3 | 90.3 | 0.29 | 0.59 | 87.9 | 91.9 | 4.74 | **0.03** | 89.6 | 90.1 | 0.05 | 0.83 | 90.0 | 78.6 | 3.88 | 0.06 |
|  | CT + TT ^c^ | 10.7 | 9.7 |  |  | 12.1 | 8.1 |  |  | 10.4 | 9.9 |  |  | 10.0 | 21.4 |  |  |
|  | C | 94.4 | 95.1 | 0.40 | 0.53 | 93.9 | 95.7 | 3.46 | 0.06 | 94.7 | 94.6 | 0.01 | 0.93 | 94.8 | 89.3 | 3.33 | 0.07 |
|  | T | 5.6 | 4.9 |  |  | 6.1 | 4.3 |  |  | 5.3 | 5.4 |  |  | 5.2 | 10.7 |  |  |

^a^ Abbreviations: HDLC, high-density lipoprotein cholesterol; LDLC, low-density lipoprotein cholesterol; SNPs, single nucleotide polymorphisms; TC, total cholesterol; TG, triglyceride.

^b^ Multiple comparisons revealed the significant difference of genotype frequencies of GA and AA between low and normal HDLC groups, *P* < 0.05.

^c^ Genotype frequencies of TT was too low and did not meet the criteria for Chi-square test, so genotype CT and TT were combined.
