# Supplementary material for: Genetic polymorphisms of superoxide dismutase 1 are associated with the serum lipid profiles of Han Chinese adults in a sexually dimorphic manner
Source: PLoS One. 2020 Jun 19;15(6):e0234716. doi: 10.1371/journal.pone.0234716 (PMC7304602; doi:10.1371/journal.pone.0234716)
Supplement: S5 Table — a Abbreviations: HDLC, high-density lipoprotein cholesterol; LDLC, low-density lipoprotein cholesterol; SNPs, single nucleotide polymorphisms; TC, total cholesterol; TG, triglyceride. b Multiple comparisons revealed the significant difference of genotype frequencies of GA and AA between high and normal TC groups, P < 0.05. c Genotype frequencies of TT was too low and did not meet the criteria for Chi-square test, so genotype CT and TT were combined. (DOCX) [file pone.0234716.s009.docx]

**S****5 Table. Genotype frequencies of three tag SNPs of superoxide dismutase 1 gene in abnormal and normal lipid groups of adult females** ^a^

|  |  | TG | |  |  | TC | |  |  | LDLC | |  |  | HDLC | |  |  |
| --- | --- | --- | --- | --- | --- | --- | --- | --- | --- | --- | --- | --- | --- | --- | --- | --- | --- |
| mmol/L | | < 1.7 | ≥ 1.7 |  |  | < 5.2 | ≥ 5.2 |  |  | < 3.4 | ≥ 3.4 |  |  | ≥ 1.0 | < 1.0 |  |  |
| *n* | | 1274 | 237 |  |  | 894 | 617 |  |  | 1226 | 285 |  |  | 1477 | 34 |  |  |
| Genotype | | % | | *χ^2^* | *P* | % | | *χ^2^* | *P* | % | | *χ^2^* | *P* | % | | *χ^2^* | *P* |
| rs4998557 | GG | 27.2 | 28.3 | 0.37 | 0.83 | 27.2 | 27.6 | 8.70 | **0.01** ^b^ | 26.6 | 30.5 | 2.19 | 0.33 | 27.2 | 32.4 | 0.72 | 0.70 |
|  | GA | 46.5 | 47.3 |  |  | 44.2 | 50.2 |  |  | 46.8 | 46.0 |  |  | 46.6 | 47.1 |  |  |
|  | AA | 26.3 | 24.5 |  |  | 28.6 | 22.2 |  |  | 26.6 | 23.5 |  |  | 26.1 | 20.6 |  |  |
|  | G | 50.4 | 51.9 | 0.34 | 0.56 | 49.3 | 52.7 | 3.38 | 0.07 | 50.0 | 53.5 | 2.28 | 0.13 | 50.5 | 55.9 | 0.76 | 0.38 |
|  | A | 49.6 | 48.1 |  |  | 50.7 | 47.3 |  |  | 50.0 | 46.5 |  |  | 49.5 | 44.1 |  |  |
| rs1041740 | CC | 43.6 | 42.6 | 0.10 | 0.95 | 42.8 | 44.4 | 2.34 | 0.31 | 43.1 | 44.9 | 0.40 | 0.82 | 43.7 | 35.3 | 2.27 | 0.32 |
|  | CT | 43.2 | 44.3 |  |  | 43.0 | 44.1 |  |  | 43.8 | 41.8 |  |  | 43.1 | 55.9 |  |  |
|  | TT | 13.1 | 13.1 |  |  | 14.2 | 11.5 |  |  | 13.1 | 13.3 |  |  | 13.2 | 8.8 |  |  |
|  | C | 65.3 | 64.8 | 0.04 | 0.83 | 64.3 | 66.5 | 1.46 | 0.23 | 65.0 | 65.8 | 0.11 | 0.74 | 65.2 | 63.2 | 0.11 | 0.73 |
|  | T | 34.7 | 35.2 |  |  | 35.7 | 33.5 |  |  | 35.0 | 34.2 |  |  | 34.8 | 36.8 |  |  |
| rs17880487 | CC | 89.0 | 89.0 | 0.01 | 0.99 | 89.0 | 89.0 | 0.01 | 0.97 | 89.4 | 87.4 | 0.97 | 0.32 | 89.2 | 82.4 | 1.58 | 0.21 |
|  | CT + TT ^c^ | 11.0 | 11.0 |  |  | 11.0 | 11.0 |  |  | 10.6 | 12.6 |  |  | 10.8 | 17.6 |  |  |
|  | C | 94.3 | 94.3 | 0.00 | 0.98 | 94.4 | 94.1 | 0.14 | 0.71 | 94.6 | 93.0 | 2.18 | 0.14 | 94.3 | 91.2 | 1.24 | 0.27 |
|  | T | 5.7 | 5.7 |  |  | 5.6 | 5.9 |  |  | 5.4 | 7.0 |  |  | 5.7 | 8.8 |  |  |

^a^ Abbreviations: HDLC, high-density lipoprotein cholesterol; LDLC, low-density lipoprotein cholesterol; SNPs, single nucleotide polymorphisms; TC, total cholesterol; TG, triglyceride.

^b^ Multiple comparisons revealed the significant difference of genotype frequencies of GA and AA between high and normal TC groups, *P* < 0.05.

^c^ Genotype frequencies of TT was too low and did not meet the criteria for Chi-square test, so genotype CT and TT were combined.
