# Supplementary material for: Genetic polymorphisms of superoxide dismutase 1 are associated with the serum lipid profiles of Han Chinese adults in a sexually dimorphic manner
Source: PLoS One. 2020 Jun 19;15(6):e0234716. doi: 10.1371/journal.pone.0234716 (PMC7304602; doi:10.1371/journal.pone.0234716)
Supplement: S6 Table — a Abbreviations: Add., additive model; Alle., allelic model; Dom., dominant model; HDLC, high-density lipoprotein cholesterol; Hom., homozygous model; IQR, interquartile range; LDLC, low-density lipoprotein cholesterol; Rec., recessive model; SNPs, single nucleotide polymorphisms; TC, total cholesterol; TG, triglyceride. b Multiple comparisons revealed the significant difference between the genotypes of CT and CC, P < 0.05. (DOCX) [file pone.0234716.s010.docx]

**S6 Table. Comparison of lipid levels across genotype models of three tag SNPs of superoxide dismutase 1 gene in adult males** ^a^

| Genotype |  |  | TG |  |  |  | TC |  |  |  | LDLC |  |  |  | HDLC |  |
| --- | --- | --- | --- | --- | --- | --- | --- | --- | --- | --- | --- | --- | --- | --- | --- | --- |
| Comparison | *n* | Median | IQR | *P* |  | Median | IQR | *P* |  | Median | IQR | *P* |  | Median | IQR | *P* |
| **rs4998557** |  |  |  |  |  |  |  |  |  |  |  |  |  |  |  |  |
| Add.: GG vs AG vs AA |  |  |  |  |  |  |  |  |  |  |  |  |  |  |  |  |
| GG | 283 | 1.39 | [0.93 - 2.15] | 0.76 |  | 5.14 | [4.45 - 5.68] | 0.46 |  | 2.88 | [2.53 - 3.29] | 0.58 |  | 1.36 | [1.23 - 1.52] | 0.52 |
| AG | 543 | 1.44 | [1.00 - 2.24] |  |  | 5.07 | [4.47 - 5.77] |  |  | 2.89 | [2.51 - 3.27] |  |  | 1.36 | [1.23 - 1.53] |  |
| AA | 284 | 1.39 | [0.94 - 2.37] |  |  | 5.02 | [4.43 - 5.60] |  |  | 2.84 | [2.48 - 3.23] |  |  | 1.34 | [1.23 - 1.50] |  |
| Dom.: AA + AG vs GG |  |  |  |  |  |  |  |  |  |  |  |  |  |  |  |  |
| AA + AG | 827 | 1.43 | [0.98 - 2.29] | 0.46 |  | 5.05 | [4.46 - 5.69] | 0.96 |  | 2.88 | [2.50 - 3.26] | 0.85 |  | 1.35 | [1.23 - 1.52] | 0.94 |
| GG | 283 | 1.39 | [0.93 - 2.15] |  |  | 5.14 | [4.45 - 5.68] |  |  | 2.88 | [2.53 - 3.29] |  |  | 1.36 | [1.23 - 1.52] |  |
| Rec.: AA vs AG + GG |  |  |  |  |  |  |  |  |  |  |  |  |  |  |  |  |
| AA | 284 | 1.39 | [0.94 - 2.37] | 0.90 |  | 5.02 | [4.43 - 5.60] | 0.25 |  | 2.84 | [2.48 - 3.23] | 0.31 |  | 1.34 | [1.23 - 1.50] | 0.27 |
| AG + GG | 826 | 1.43 | [0.96 - 2.23] |  |  | 5.11 | [4.47 - 5.71] |  |  | 2.89 | [2.52 - 3.27] |  |  | 1.36 | [1.23 - 1.52] |  |
| Hom.: AA vs GG |  |  |  |  |  |  |  |  |  |  |  |  |  |  |  |  |
| AA | 284 | 1.39 | [0.94 - 2.37] | 0.58 |  | 5.02 | [4.43 - 5.60] | 0.53 |  | 2.84 | [2.48 - 3.23] | 0.50 |  | 1.34 | [1.23 - 1.50] | 0.52 |
| GG | 283 | 1.39 | [0.93 - 2.15] |  |  | 5.14 | [4.45 - 5.68] |  |  | 2.88 | [2.53 - 3.29] |  |  | 1.36 | [1.23 - 1.52] |  |
| Alle.: A vs G |  |  |  |  |  |  |  |  |  |  |  |  |  |  |  |  |
| A | 1111 | 1.42 | [0.96 - 2.33] | 0.59 |  | 5.04 | [4.45 - 5.67] | 0.50 |  | 2.87 | [2.50 - 3.25] | 0.45 |  | 1.35 | [1.23 - 1.51] | 0.47 |
| G | 1109 | 1.43 | [0.95 - 2.21] |  |  | 5.12 | [4.47 - 5.70] |  |  | 2.89 | [2.52 - 3.27] |  |  | 1.36 | [1.23 - 1.52] |  |
| **rs1041740** |  |  |  |  |  |  |  |  |  |  |  |  |  |  |  |  |
| Add.: CC vs CT vs TT |  |  |  |  |  |  |  |  |  |  |  |  |  |  |  |  |
| CC | 475 | 1.44 | [0.98 - 2.23] | 0.76 |  | 5.14 | [4.47 - 5.76] | 0.86 |  | 2.89 | [2.49 - 3.28] | 0.57 |  | 1.36 | [1.23 - 1.53] | 0.61 |
| CT | 508 | 1.41 | [0.95 - 2.25] |  |  | 5.03 | [4.48 - 5.61] |  |  | 2.86 | [2.52 - 3.21] |  |  | 1.35 | [1.23 - 1.51] |  |
| TT | 127 | 1.43 | [0.94 - 2.47] |  |  | 5.16 | [4.38 - 5.74] |  |  | 2.93 | [2.50 - 3.36] |  |  | 1.36 | [1.26 - 1.50] |  |

**(Continued) S6 Table.**

| Genotype |  |  | TG |  |  |  | TC |  |  |  | LDLC |  |  |  | HDLC |  |
| --- | --- | --- | --- | --- | --- | --- | --- | --- | --- | --- | --- | --- | --- | --- | --- | --- |
| Comparison | *n* | Median | IQR | *P* |  | Median | IQR | *P* |  | Median | IQR | *P* |  | Median | IQR | *P* |
| Dom.: CT + TT vs CC |  |  |  |  |  |  |  |  |  |  |  |  |  |  |  |  |
| CT + TT | 635 | 1.41 | [0.95 - 2.29] | 0.89 |  | 5.03 | [4.45 - 5.66] | .0.61 |  | 2.87 | [2.52 - 3.25] | 0.77 |  | 1.35 | [1.23 - 1.51] | 0.37 |
| CC | 475 | 1.44 | [0.98 - 2.23] |  |  | 5.14 | [4.47 - 5.76] |  |  | 2.89 | [2.49 - 3.28] |  |  | 1.36 | [1.23 - 1.53] |  |
| Rec.: TT vs CT + CC |  |  |  |  |  |  |  |  |  |  |  |  |  |  |  |  |
| TT | 127 | 1.43 | [0.94 - 2.47] | 0.52 |  | 5.16 | [4.38 - 5.74] | 0.95 |  | 2.93 | [2.50 - 3.36] | 0.38 |  | 1.36 | [1.26 - 1.50] | 0.90 |
| CT + CC | 983 | 1.42 | [0.96 - 2.24] |  |  | 5.07 | [4.48 - 5.68] |  |  | 2.87 | [2.51 - 3.25] |  |  | 1.36 | [1.23 - 1.52] |  |
| Hom.: TT vs CC |  |  |  |  |  |  |  |  |  |  |  |  |  |  |  |  |
| TT | 127 | 1.43 | [0.94 - 2.47] | 0.59 |  | 5.16 | [4.38 - 5.74] | 0.93 |  | 2.93 | [2.50 - 3.36] | 0.51 |  | 1.36 | [1.26 - 1.50] | 0.83 |
| CC | 475 | 1.44 | [0.98 - 2.23] |  |  | 5.14 | [4.47 - 5.76] |  |  | 2.89 | [2.49 - 3.28] |  |  | 1.36 | [1.23 - 1.53] |  |
| Alle.: T vs C |  |  |  |  |  |  |  |  |  |  |  |  |  |  |  |  |
| T | 762 | 1.42 | [0.95 - 2.30] | 0.84 |  | 5.04 | [4.43 - 5.67] | 0.74 |  | 2.88 | [2.52 - 3.27] | 0.84 |  | 1.35 | [1.24 - 1.50] | 0.55 |
| C | 1458 | 1.43 | [0.96 - 2.23] |  |  | 5.10 | [4.48 - 5.70] |  |  | 2.88 | [2.50 - 3.26] |  |  | 1.36 | [1.23 - 1.52] |  |
| **rs17880487** |  |  |  |  |  |  |  |  |  |  |  |  |  |  |  |  |
| Add.: CC vs CT vs TT |  |  |  |  |  |  |  |  |  |  |  |  |  |  |  |  |
| CC | 996 | 1.43 | [0.97 - 2.24] | 0.91 |  | 5.10 | [4.48 - 5.69] | **0.04** ^b^ |  | 2.89 | [2.25 - 3.26] | **0.047** ^b^ |  | 1.36 | [1.23 - 1.52] | 0.26 |
| CT | 110 | 1.40 | [0.88 - 2.41] |  |  | 4.87 | [4.22 - 5.50] |  |  | 2.71 | [2.36 - 3.20] |  |  | 1.33 | [1.17 - 1.50] |  |
| TT | 4 | 1.57 | [0.92 - 1.92] |  |  | 5.70 | [4.47 - 6.58] |  |  | 3.17 | [2.36 - 3.77] |  |  | 1.52 | [1.14 - 1.68] |  |
| Dom.: CT + TT vs CC |  |  |  |  |  |  |  |  |  |  |  |  |  |  |  |  |
| CT + TT | 114 | 1.41 | [0.88 - 2.37] | 0.67 |  | 4.92 | [4.22 - 5.51] | **0.04** |  | 2.74 | [2.36 - 3.22] | **0.03** |  | 1.34 | [1.17-1.50] | 0.21 |
| CC | 996 | 1.43 | [0.97 - 2.24] |  |  | 5.10 | [4.48 - 5.69] |  |  | 2.89 | [2.25 - 3.26] |  |  | 1.36 | [1.23 - 1.52] |  |

**(Continued) S6 Table.**

| Genotype |  |  | TG |  |  |  | TC |  |  |  | LDLC |  |  |  | HDLC |  |
| --- | --- | --- | --- | --- | --- | --- | --- | --- | --- | --- | --- | --- | --- | --- | --- | --- |
| Comparison | *n* | Median | IQR | *P* |  | Median | IQR | *P* |  | Median | IQR | *P* |  | Median | IQR | *P* |
| Rec.: TT vs CT + CC |  |  |  |  |  |  |  |  |  |  |  |  |  |  |  |  |
| TT | 4 | 1.57 | [0.92 - 1.92] | 0.90 |  | 5.70 | [4.47 - 6.58] | 0.30 |  | 3.17 | [2.36 - 3.77] | 0.52 |  | 1.52 | [1.14 - 1.68] | 0.42 |
| CT + CC | 1106 | 1.42 | [0.96 - 2.24] |  |  | 5.07 | [4.46 - 5.68] |  |  | 2.88 | [2.51 - 3.26] |  |  | 1.36 | [1.23 - 1.52] |  |
| Hom.: TT vs CC |  |  |  |  |  |  |  |  |  |  |  |  |  |  |  |  |
| TT | 4 | 1.57 | [0.92 - 1.92] | 0.89 |  | 5.70 | [4.47 - 6.58] | 0.31 |  | 3.17 | [2.36 - 3.77] | 0.54 |  | 1.52 | [1.14 - 1.68] | 0.42 |
| CC | 996 | 1.44 | [1.00 - 2.24] |  |  | 5.07 | [4.47 - 5.77] |  |  | 2.89 | [2.51 - 3.27] |  |  | 1.36 | [1.23 - 1.53] |  |
| Alle.: T vs C |  |  |  |  |  |  |  |  |  |  |  |  |  |  |  |  |
| T | 118 | 1.43 | [0.88 - 2.30] | 0.67 |  | 4.95 | [4.22 – 5.59] | 0.07 |  | 2.75 | [2.36 - 3.27] | **0.04** |  | 1.35 | [1.17 - 1.51] | 0.30 |
| C | 2102 | 1.43 | [0.96 - 2.24] |  |  | 5.08 | [4.47 - 5.69] |  |  | 2.88 | [2.52 - 3.26] |  |  | 1.36 | [1.23 - 1.52] |  |

^a^ Abbreviations: Add., additive model; Alle., allelic model; Dom., dominant model; HDLC, high-density lipoprotein cholesterol; Hom., homozygous model; IQR, interquartile range; LDLC, low-density lipoprotein cholesterol; Rec., recessive model; SNPs, single nucleotide polymorphisms; TC, total cholesterol; TG, triglyceride.

^b^ Multiple comparisons revealed the significant difference between the genotypes of CT and CC, *P* < 0.05.
