# Supplementary material for: Genetic polymorphisms of superoxide dismutase 1 are associated with the serum lipid profiles of Han Chinese adults in a sexually dimorphic manner
Source: PLoS One. 2020 Jun 19;15(6):e0234716. doi: 10.1371/journal.pone.0234716 (PMC7304602; doi:10.1371/journal.pone.0234716)
Supplement: S7 Table — a Abbreviations: Add., additive model; Alle., allelic model; Dom., dominant model; HDLC, high-density lipoprotein cholesterol; Hom., homozygous model; IQR, interquartile range; LDLC, low-density lipoprotein cholesterol; Rec., recessive model; SNPs, single nucleotide polymorphisms; TC, total cholesterol; TG, triglyceride. (DOCX) [file pone.0234716.s011.docx]

**S7 Table.** **Comparison of lipid levels across genotype models of three tag SNPs of superoxide dismutase 1 gene in adult females** ^a^

| Genotype |  |  | TG |  |  |  | TC |  |  |  | LDLC |  |  |  | HDLC |  |
| --- | --- | --- | --- | --- | --- | --- | --- | --- | --- | --- | --- | --- | --- | --- | --- | --- |
| Comparison | *n* | Median | IQR | *P* |  | Median | IQR | *P* |  | Median | IQR | *P* |  | Median | IQR | *P* |
| **rs4998557** |  |  |  |  |  |  |  |  |  |  |  |  |  |  |  |  |
| Add: GG vs AG vs AA |  |  |  |  |  |  |  |  |  |  |  |  |  |  |  |  |
| GG | 413 | 0.85 | [0.59 - 1.35] | 0.64 |  | 4.97 | [4.40 - 5.70] | 0.30 |  | 2.83 | [2.45 - 3.27] | 0.29 |  | 1.46 | [1.29 - 1.61] | 0.97 |
| AG | 705 | 0.89 | [0.63 - 1.40] |  |  | 5.04 | [4.43 - 5.67] |  |  | 2.82 | [2.46 - 3.23] |  |  | 1.44 | [1.28 - 1.62] |  |
| AA | 393 | 0.88 | [0.59 - 1.32] |  |  | 4.94 | [4.37 - 5.47] |  |  | 2.76 | [2.44 - 3.16] |  |  | 1.44 | [1.27 - 1.62] |  |
| Dom.: AA + AG vs GG |  |  |  |  |  |  |  |  |  |  |  |  |  |  |  |  |
| AA + AG | 1098 | 0.89 | [0.62 - 1.36] | 0.43 |  | 4.98 | [4.41 - 5.61] | 0.91 |  | 2.97 | [2.44 - 3.21] | 0.32 |  | 1.44 | [1.23 - 1.52] | 0.90 |
| GG | 413 | 0.85 | [0.59 - 1.35] |  |  | 4.97 | [4.40 - 5.70] |  |  | 2.83 | [2.45 - 3.27] |  |  | 1.46 | [1.28 - 1.62] |  |
| Rec.: AA vs AG + GG |  |  |  |  |  |  |  |  |  |  |  |  |  |  |  |  |
| AA | 393 | 0.88 | [0.59 - 1.32] | 0.86 |  | 4.94 | [4.37 - 5.47] | 0.14 |  | 2.76 | [2.44 - 3.16] | 0.13 |  | 1.44 | [1.27 - 1.62] | 0.80 |
| AG + GG | 1118 | 0.87 | [0.62 - 1.38] |  |  | 5.00 | [4.41 - 5.57] |  |  | 2.82 | [2.45 - 3.25] |  |  | 1.45 | [1.28 - 1.62] |  |
| Hom.: AA vs GG |  |  |  |  |  |  |  |  |  |  |  |  |  |  |  |  |
| AA | 393 | 0.88 | [0.59 - 1.32] | 0.80 |  | 4.94 | [4.37 - 5.47] | 0.33 |  | 2.76 | [2.44 - 3.16] | 0.13 |  | 1.44 | [1.27 - 1.62] | 0.76 |
| GG | 413 | 0.85 | [0.59 - 1.35] |  |  | 4.97 | [4.40 - 5.70] |  |  | 2.83 | [2.45 - 3.27] |  |  | 1.46 | [1.29 - 1.61] |  |
| Alle,: A vs G |  |  |  |  |  |  |  |  |  |  |  |  |  |  |  |  |
| A | 1491 | 0.88 | [0.61 - 1.35] | 0.70 |  | 4.96 | [4.39 - 5.58] | 0.32 |  | 2.78 | [2.44 - 3.20] | 0.12 |  | 1.44 | [1.28 - 1.62] | 0.81 |
| G | 1531 | 0.87 | [0.61 - 1.36] |  |  | 4.98 | [4.40 - 5.68] |  |  | 2.82 | [2.45 - 3.25] |  |  | 1.45 | [1.28 - 1.62] |  |
| **rs1041740** |  |  |  |  |  |  |  |  |  |  |  |  |  |  |  |  |
| Add.: CC vs CT vs TT |  |  |  |  |  |  |  |  |  |  |  |  |  |  |  |  |
| CC | 657 | 0.87 | [0.61 - 1.34] | 0.91 |  | 4.97 | [4.39 - 5.69] | 0.51 |  | 2.82 | [2.45 - 3.24] | 0.25 |  | 1.45 | [1.29 - 1.61] | 0.93 |
| CT | 656 | 0.88 | [0.62 - 1.38] |  |  | 5.01 | [4.43 - 5.60] |  |  | 2.82 | [2.46 - 3.24] |  |  | 1.45 | [1.28 - 1.63] |  |
| TT | 198 | 0.89 | [0.59 - 1.35] |  |  | 4.92 | [4.37 - 5.54] |  |  | 2.69 | [2.42 - 3.21] |  |  | 1.45 | [1.26 - 1.62] |  |

**(Continued) S7 Table.**

| Genotype |  |  | TG |  |  |  | TC |  |  |  | LDLC |  |  |  | HDLC |  |
| --- | --- | --- | --- | --- | --- | --- | --- | --- | --- | --- | --- | --- | --- | --- | --- | --- |
| Comparison | *n* | Median | IQR | *P* |  | Median | IQR | *P* |  | Median | IQR | *P* |  | Median | IQR | *P* |
| Dom.: CT + TT vs CC |  |  |  |  |  |  |  |  |  |  |  |  |  |  |  |  |
| CT + TT | 854 | 0.88 | [0.62 - 1.37] | 0.73 |  | 4.98 | [4.42 - 5.59] | 0.99 |  | 2.79 | [2.44 - 3.23] | 0.64 |  | 1.45 | [1.28 - 1.63] | 0.58 |
| CC | 657 | 0.87 | [0.61 - 1.34] |  |  | 4.97 | [4.39 - 5.69] |  |  | 2.82 | [2.45 - 3.24] |  |  | 1.45 | [1.29 - 1.61] |  |
| Rec.: TT vs CT + CC |  |  |  |  |  |  |  |  |  |  |  |  |  |  |  |  |
| TT | 198 | 0.89 | [0.59 - 1.35] | 0.89 |  | 4.92 | [4.37 - 5.54] | 0.27 |  | 2.69 | [2.42 - 3.21] | 0.10 |  | 1.45 | [1.26 - 1.62] | 0.72 |
| CT + CC | 1313 | 0.87 | [0.62 - 1.36] |  |  | 4.98 | [4.41 - 5.65] |  |  | 2.82 | [2.45 - 3.24] |  |  | 1.45 | [1.29 - 1.62] |  |
| Hom.: TT vs CC |  |  |  |  |  |  |  |  |  |  |  |  |  |  |  |  |
| TT | 198 | 0.89 | [0.59 - 1.35] | 0.99 |  | 4.92 | [4.37 - 5.54] | 0.39 |  | 2.69 | [2.42 - 3.21] | 0.14 |  | 1.45 | [1.26 - 1.62] | 0.91 |
| CC | 657 | 0.87 | [0.61 - 1.34] |  |  | 4.97 | [4.39 - 5.69] |  |  | 2.82 | [2.45 - 3.24] |  |  | 1.45 | [1.29 - 1.61] |  |
| Alle.: T vs C |  |  |  |  |  |  |  |  |  |  |  |  |  |  |  |  |
| T | 1052 | 0.88 | [0.60 - 1.36] | 0.86 |  | 4.96 | [4.40 - 5.58] | 0.57 |  | 2.77 | [2.44 - 3.23] | 0.24 |  | 1.45 | [1.27 - 1.63] | 0.82 |
| C | 1970 | 0.87 | [0.61 - 1.35] |  |  | 4.98 | [4.40 - 5.66] |  |  | 2.82 | [2.45 - 3.24] |  |  | 1.45 | [1.29 - 1.61] |  |
| **rs17880487** |  |  |  |  |  |  |  |  |  |  |  |  |  |  |  |  |
| Add.: CC vs CT vs TT |  |  |  |  |  |  |  |  |  |  |  |  |  |  |  |  |
| CC | 1345 | 0.88 | [0.61 - 1.36] | 0.19 |  | 4.98 | [4.42 - 5.62] | 0.26 |  | 2.81 | [2.45 - 3.23] | 0.74 |  | 1.45 | [1.28 - 1.68] | 0.40 |
| CT | 159 | 0.82 | [0.62 - 1.33] |  |  | 4.90 | [4.27 - 5.75] |  |  | 2.75 | [2.40 - 3.27] |  |  | 1.43 | [1.29 - 1.59] |  |
| TT | 7 | 1.28 | [0.92-1.57] |  |  | 5.96 | [4.73 - 6.35] |  |  | 3.52 | [2.54 - 3.73] |  |  | 1.48 | [1.41 - 1.68] |  |
| Dom.: CT + TT vs CC |  |  |  |  |  |  |  |  |  |  |  |  |  |  |  |  |
| CT + TT | 166 | 0.83 | [0.64 - 1.33] | 0.57 |  | 4.90 | [4.28 - 5.78] | 0.71 |  | 2.76 | [2.41 - 3.29] | 0.55 |  | 1.43 | [1.30 - 1.59] | 0.64 |
| CC | 1345 | 0.88 | [0.61 - 1.36] |  |  | 4.98 | [4.42 - 5.62] |  |  | 2.81 | [2.45 - 3.23] |  |  | 1.45 | [1.28 - 1.68] |  |

**(Continued) S7 Table.**

| Genotype |  |  | TG |  |  |  | TC |  |  |  | LDLC |  |  |  | HDLC |  |
| --- | --- | --- | --- | --- | --- | --- | --- | --- | --- | --- | --- | --- | --- | --- | --- | --- |
| Comparison | *n* | Median | IQR | *P* |  | Median | IQR | *P* |  | Median | IQR | *P* |  | Median | IQR | *P* |
| Rec.: TT vs CT + CC |  |  |  |  |  |  |  |  |  |  |  |  |  |  |  |  |
| TT | 7 | 1.28 | [0.92-1.57] | 0.11 |  | 5.96 | [4.73 - 6.35] | 0.14 |  | 3.52 | [2.54 - 3.73] | 0.18 |  | 1.48 | [1.41 - 1.68] | 0.25 |
| CT + CC | 1504 | 0.87 | [0.61 - 1.35] |  |  | 4.97 | [4.40 - 5.63] |  |  | 2.81 | [2.44 - 3.23] |  |  | 1.45 | [1.28 - 1.62] |  |
| Hom.: TT vs CC |  |  |  |  |  |  |  |  |  |  |  |  |  |  |  |  |
| TT | 7 | 1.28 | [0.92 - 1.57] | 0.12 |  | 5.96 | [4.73 - 6.35] | 0.14 |  | 3.52 | [2.54 - 3.73] | 0.18 |  | 1.48 | [1.41 - 1.68] | 0.25 |
| CC | 1345 | 0.88 | [0.61 - 1.36] |  |  | 4.98 | [4.42 - 5.62] |  |  | 2.81 | [2.45 - 3.23] |  |  | 1.45 | [1.28 - 1.68] |  |
| Alle.: T vs C |  |  |  |  |  |  |  |  |  |  |  |  |  |  |  |  |
| T | 173 | 0.84 | [0.64 - 1.33] | 0.83 |  | 4.97 | [4.28 - 5.83] | 0.96 |  | 2.78 | [2.42 - 3.33] | 0.77 |  | 1.44 | [1.30 - 1.60] | 0.83 |
| C | 2849 | 0.88 | [0.61 - 1.36] |  |  | 4.97 | [4.41 - 5.63] |  |  | 2.81 | [2.45 - 3.23] |  |  | 1.45 | [1.28 - 1.62] |  |

^a^ Abbreviations: Add., additive model; Alle., allelic model; Dom., dominant model; HDLC, high-density lipoprotein cholesterol; Hom., homozygous model; IQR, interquartile range; LDLC, low-density lipoprotein cholesterol; Rec., recessive model; SNPs, single nucleotide polymorphisms; TC, total cholesterol; TG, triglyceride.
