# Supplementary material for: Genetic polymorphisms of superoxide dismutase 1 are associated with the serum lipid profiles of Han Chinese adults in a sexually dimorphic manner
Source: PLoS One. 2020 Jun 19;15(6):e0234716. doi: 10.1371/journal.pone.0234716 (PMC7304602; doi:10.1371/journal.pone.0234716)
Supplement: Attachment — Submitted filename: Comments-Responses-200506.docx [file pone.0234716.s012.docx]

Dear editor and reviewers,

Thank you very much for your critical and constructive comments!

Attention please: all the underlined text in this word file are from the authors, otherwise from editor, reviewer, or PloS ONE processing system.

PONE-D-19-26611

Genetic polymorphisms of superoxide dismutase 1 are associated with the serum lipid profiles of Han Chinese adults in a sexually dimorphic manner

PLOS ONE

Dear Dr. Zhou,

Thank you for submitting your manuscript to PLOS ONE. After careful consideration, we feel that it has merit but does not fully meet PLOS ONE’s publication criteria as it currently stands. Therefore, we invite you to submit a revised version of the manuscript that addresses the points raised during the review process.

The manuscript has been evaluated by two reviewers, and their comments are available below.

The reviewers have raised a number of major concerns. They feel the manuscript should outline a clearly-defined research question, and they request improvements to the reporting of methodological aspects of the study, for example, regarding the sequencing and statistical analyses. In addition, terms such as "mechanical correlations" and “certain inclusion criteria“ should be described in more detail.

Re: Thanks for the comments. We improved the writing of the introduction to make the research question clearer as well as the methodological aspects including sequencing and statistical analyses.

We misused the "mechanical correlations" to express the meaning of mechanism roles and corrected as “mechanistic correlations". As described in the Introduction section, it was found that knockout/inhibition of SOD1 induced disorders of lipid metabolism and gender generally played roles in many diseases, and therefore we want to explore the association of *SOD1* SNPs and lipid parameters considering the gender difference via epidemiologic studies, which has not been reported.

In the abstract, we replaced “with certain inclusion criteria” with “without organic diseases, cancers, and pregnancy” which was explained in the main text of the Participants and sample collection section.

Could you please carefully revise the manuscript to address all comments raised?

Re: Yes, we did as suggested or requested.

We would appreciate receiving your revised manuscript by May 04 2020 11:59PM. When you are ready to submit your revision, log on to https://www.editorialmanager.com/pone/ and select the 'Submissions Needing Revision' folder to locate your manuscript file.

If you would like to make changes to your financial disclosure, please include your updated statement in your cover letter.

To enhance the reproducibility of your results, we recommend that if applicable you deposit your laboratory protocols in protocols.io, where a protocol can be assigned its own identifier (DOI) such that it can be cited independently in the future. For instructions see: http://journals.plos.org/plosone/s/submission-guidelines#loc-laboratory-protocols

Please include the following items when submitting your revised manuscript:

•A rebuttal letter that responds to each point raised by the academic editor and reviewer(s). This letter should be uploaded as separate file and labeled 'Response to Reviewers'.

•A marked-up copy of your manuscript that highlights changes made to the original version. This file should be uploaded as separate file and labeled 'Revised Manuscript with Track Changes'.

•An unmarked version of your revised paper without tracked changes. This file should be uploaded as separate file and labeled 'Manuscript'.

Please note while forming your response, if your article is accepted, you may have the opportunity to make the peer review history publicly available. The record will include editor decision letters (with reviews) and your responses to reviewer comments. If eligible, we will contact you to opt in or out.

We look forward to receiving your revised manuscript.

Kind regards,

Natasha Rickett

Academic Editor

PLOS ONE

Journal Requirements:

When submitting your revision, we need you to address these additional requirements:

1. Please ensure that your manuscript meets PLOS ONE's style requirements, including those for file naming. The PLOS ONE style templates can be found at http://www.plosone.org/attachments/PLOSOne_formatting_sample_main_body.pdf and http://www.plosone.org/attachments/PLOSOne_formatting_sample_title_authors_affiliations.pdf

Re: Yes, we follow the journal’s style as far as we know.

2. We suggest you thoroughly copyedit your manuscript for language usage, spelling, and grammar. If you do not know anyone who can help you do this, you may wish to consider employing a professional scientific editing service.

Re: Yes, we have thoroughly copyedit our manuscript.

Whilst you may use any professional scientific editing service of your choice, PLOS has partnered with both American Journal Experts (AJE) and Editage to provide discounted services to PLOS authors. Both organizations have experience helping authors meet PLOS guidelines and can provide language editing, translation, manuscript formatting, and figure formatting to ensure your manuscript meets our submission guidelines. To take advantage of our partnership with AJE, visit the AJE website (http://learn.aje.com/plos/) for a 15% discount off AJE services. To take advantage of our partnership with Editage, visit the Editage website (www.editage.com) and enter referral code PLOSEDIT for a 15% discount off Editage services. If the PLOS editorial team finds any language issues in text that either AJE or Editage has edited, the service provider will re-edit the text for free.

Re: Thank you for your kindness to provide this useful information, but beside article processing charge, we do not have budget for editing service. Thus, we have tried our best to check throughout the manuscript for language and typo issues.

Upon resubmission, please provide the following:

•The name of the colleague or the details of the professional service that edited your manuscript

•A copy of your manuscript showing your changes by either highlighting them or using track changes (uploaded as a *supporting information* file)

•A clean copy of the edited manuscript (uploaded as the new *manuscript* file)

3. Please refer to any post-hoc corrections to correct for multiple comparisons during your statistical analyses. if these were not performed please justify the reasons. Also, please ensure you have thoroughly described your methodologies to the standard that they could be repeated. Phrases such as the blood being collected in "tubes with or without EDTA anticoagulation" create confusion.

Re: Multiple comparisons were performed when comparison groups were ≥ 3 with significant differences, as shown below the S4-S6 tables**.** We have checked the methodologies carefully, and it is guaranteed the methodologies can be repeated. Two blood samples were collected from each participant, and one was drawn into a vacuum tube with EDTA anticoagulant and the other was into a vacuum tube without any anticoagulants, which was between lines 101-103 in the revised clean main text.

[Note: HTML markup is below. Please do not edit.]

Reviewers' comments:

Reviewer's Responses to Questions

Comments to the Author

1. Is the manuscript technically sound, and do the data support the conclusions?

The manuscript must describe a technically sound piece of scientific research with data that supports the conclusions. Experiments must have been conducted rigorously, with appropriate controls, replication, and sample sizes. The conclusions must be drawn appropriately based on the data presented.

Reviewer #1: Yes

Reviewer #2: No

Re: We followed the rules to conduct the experiment and supplemented some neglected description for the technically sound piece in the revised Materials and methods section.

2. Has the statistical analysis been performed appropriately and rigorously?

Reviewer #1: Yes

Reviewer #2: No

Re: In the response to Reviewer #2’s comment about the issues later, we explained the reason why we converted the continuous parameters of lipid concentrations into binary parameters, and also analyzed these non-normally distributed continuous parameters in S6-S7 Table.

3. Have the authors made all data underlying the findings in their manuscript fully available?

The PLOS Data policy requires authors to make all data underlying the findings described in their manuscript fully available without restriction, with rare exception (please refer to the Data Availability Statement in the manuscript PDF file). The data should be provided as part of the manuscript or its supporting information, or deposited to a public repository. For example, in addition to summary statistics, the data points behind means, medians and variance measures should be available. If there are restrictions on publicly sharing data—e.g. participant privacy or use of data from a third party—those must be specified.

Reviewer #1: Yes

Reviewer #2: Yes

4. Is the manuscript presented in an intelligible fashion and written in standard English?

PLOS ONE does not copyedit accepted manuscripts, so the language in submitted articles must be clear, correct, and unambiguous. Any typographical or grammatical errors should be corrected at revision, so please note any specific errors here.

Reviewer #1: Yes

Reviewer #2: No

Re: We checked the language issues for standard English as far as we can.

5. Review Comments to the Author

Please use the space provided to explain your answers to the questions above. You may also include additional comments for the author, including concerns about dual publication, research ethics, or publication ethics. (Please upload your review as an attachment if it exceeds 20,000 characters)

Reviewer #1: The articles by Xu et al. describe the SOD1 polymorphisms with the lipid disorders in the adult males and females of the Chinese.

This article is of interest but some points should be clarified:

Page 2, line 60: Is enough that the patients living in Shenzhen for > 2 years. This point is very important because of life style and it should be influenced in the results.

Re: Considering Shenzhen being a modern city for many young migrants, we set the living time of > 2 years for the participant candidates originally. But in fact, most of the participants recruited from the health examination center were not that young as manifested by the age data in Table 1, and most of them had lived in the city for > 5 years. The percentage of recruited younger participants was small (those of ≤ 25 years old accounted 6.2% for males and 8.1% for females), and moreover, the young people accommodated themselves much easier after 2 years’ living in the city and had limited effect on the statistical analysis of lipid profiles.

Page 2, line 61: How the authors demonstrated that the patients were free of liver diseases, renal diseases, and any cancers in the past 6 months. Please clarify this idea in this section.

Re: The information about health conditions were collected with face to face questionnaires as well as their medical records archived in the health examination center. We explained this point in the revised manuscript.

Page 5, line 107: The authors must be described sequencing analyses.

Re: To demonstrate our genotyping method being accurate, the DNA fragments amplified and genotyped with the molecular beacon probe-based qPCR were sent to a commercial company for Sanger sequencing (gold standard method for sequence analysis) initiating by one of our qPCR primer (indicated in S1 table). The description was supplemented in the Materials and methods section and the results were presented in the revised S2-4 Fig correspondingly. Once our molecular beacon probe-based qPCR method was confirmed, all the genotyping work was performed with the method which was described in the manuscript.

Page 5, line 113: The authors must be added the OR value for the logistic regression analyses.

Re: Here we described the statistical analysis method and the OR values were listed in Table 2-3 in the Results section.

Page 5, section Data analysis: Some variable are showed in results, however it not describe in this section.

Re: We have explained these variables in section Data analysis of the revised manuscript.

Page 5, line 118: The authors must be clarified the selection of males and female patients in this study. Please include this idea in the text.

Re: Thanks for the kind reminding. The numbers of male and female patients was calculated based on the estimated sample size and a gender ratio close to 1, and the criteria for selection of males and female was almost the same as the inclusion criteria described in section Participants and sample collection. In fact, we luckily recruited and included more participants than the calculated numbers.

Discussion section: Dyslipidemia is one of the most prevalent health problems in the modern era, and multiple factors are thought to be the etiology. In this study others etiology were evaluated?. The activity of 3-hydroxy-3-methylglutaryl CoA reductase was determined?. This point is very important due to these factors could be surrogate variable. Please clarify this idea

Re: In order to explore the association between genetic polymorphisms of *SOD1* and the lipid profiles, others etiology, such as age, BMI, fasting plasma glucose, smoking status and so forth, have been listed as adjustment factors. Thank the reviewer’s suggestion, and we would chose more closely related etiological factors than the above in the future study.

Being the rate-limiting enzyme in the synthesis of cholesterol, 3-hydroxy-3-methylglutaryl CoA reductase has its high expression/activity in internal organs like liver and was almost impossible to be tested by using blood sample for the general population in the routine health examination. Moreover, we have not find a right method or kit for that purpose yet.

Thank the reviewer reminding us to consider the surrogate variables in this kind of study.

Reviewer #2: Abstract:

• Please explain the term "mechanical correlations".

• Please explain „certain inclusion criteria“.

Re: It was a typo for "mechanical correlations", and we corrected it as “mechanistic correlations" to express the meaning of correlations involving mechanism roles of SOD1 in lipid metabolism which was interpreted in detail in the introduction of the manuscript. Also in the abstract, we revised “with certain inclusion criteria” as “without organic diseases, cancers, and pregnancy” which was explained in the main text of the section of Participants and sample collection.

Participants and sample collection:

• „The people visiting the health examination center were fasted overnight and did not take medicines to control the previously diagnosed dyslipidemia for more than 12 hours“.

The effects of most lipid-lowering agents, such as statins, persist for many days. I am afraid that 12 hours without lipid-lowering medication is definitely too short.

Re: Thank the reviewer helping us to clarify this point.

Short-acting statins, such as fluvastatin, lovastatin, pravastatin, and simvastatin, have short half-lives usually less than 6 hours. Long-acting statins such as rosuvastatin and atorvastatin have half-life of 19h and 14h, respectively. Though we informed those who would come to the center for medical examination via email, phone call, or short message that we were recruiting subjects for our study, we were not sure who were really willing to participate our project before we met them and what kind of medicine they were taking. Since it was not humanitarian for us to ask the candidates who were patients not to take their regular medicine for a long time, we only reminded the candidates to suspend lipid-lowering medication overnight (> 12 h) if they were patients, and excluded those who took long-term effect medicine after we had the face-to-face questionnaire to confirm the type of medicine. This point was amended in the section of Participants and sample collection in the revised manuscript.

Results:

• The separation of the study participants into non-dyslipidemia and dyslipidemia groups does not make sense (Table 1). I would suggest to make a „lipid-lowering treatment group“ and a „no lipid-lowering treatment group“, and analyze associations of *SOD1* SNPs with lipid parameters within these groups.

Re: As shown in S2 Table, from the questionnaire survey, only 138 males (12.4%) and 95 (6.3%) females were previously diagnosed dyslipidemia patients who took medications before we asked them to suspend the medications overnight, since those taking long-term effect medicines were excluded. In addition, most of the participants revealed by our study to have unhealthy circulating lipid levels “did not know” their lipid conditions before and thus did not take medications. There was also possibility that those who thought their abnormal lipid profiles were not serious enough to have medications. If these subjects were included in no lipid-lowering treatment group, they would interfere with the association of *SOD1* SNPs with lipid parameters. Thus, we grouped those had any of the lipid parameters abnormal found in our health examination and those who were previously diagnosed patients as the dyslipidemia group (in lines 111-113 of the revised clean main text), or otherwise as the non-dyslipidemia group.

Anyway, the reviewer raised a very constructive suggestion for the grouping method for an interesting issue.

• Reducing continous parameters (e.g. cholesterol level) to binary parameters (Hypercholesterolemia yes/no) should not be done. This diminishes the statistical Power and complicates the evaluation of SOD1 allele effects (Table S4 and S5).

• Logistic regression analysis should be used for categorial outcome (e.g. dead/alive). I must not be used for metric variables such as cholesterol levels.

Re: At the beginning, we considered doing multiple linear regression to reveal the relationship between lipids levels and SOD1 SNPs, however, there was no linear relationship between blood lipids and SOD1 SNPs. By referring the similar kind of published papers (e.g. “Association of genetic polymorphisms in SOD2, SOD3, GPX3, and GSTT1 with hypertriglyceridemia and low HDL-C level in subjects with high risk of coronary artery disease”; “Gender-modulated Impact of Apolipoprotein A5 Gene (APOA5) -1131T>C and c.56C>G Polymorphisms on Lipids, Dyslipidemia and Metabolic Syndrome in Turkish Adults”; “Association of PPARα gene polymorphisms and lipid serum levels in a Brazilian elderly population”; and so forth), we decided to perform regression analysis after converting blood lipid profiles (e.g. cholesterol levels) from continuous variable to binary variable (e.g. the subjects had cholesterol level ≥ 5.2 mmol/L defined as high cholesterol). Indeed, the conversion of continuous parameters to binary parameters impairs the statistical Power, but as we all know, there is a normal range of lipid profiles, within which the clinical diagnostic criteria for dyslipidemia cannot be reached. So this conversion to binary variable made blood lipid files clinically significant and could explore the association of *SOD1* SNPs with the occurrence of dyslipidemia. As a supplement, medians and interquartile ranges for the non-normally distributed data were used to describe and compare the levels of blood lipid parameters in genotype models of the three *SOD1* SNPs (S6 Table and S7 Table). As mentioned above, lipid profiles have normal range, so this method can compare the level of blood lipid between different *SOD1* SNPs, but it is impossible to determine the relationship between *SOD1* SNPs and dyslipidemia. Moreover, logistic regression can identify whether a genotype is a risk factor or a protective factor for dyslipidemia, which we preferred. In Table S4 and S5, Chi-square test was used to find the difference in genotype distribution of the three tag SNPs of *SOD1* in abnormal and normal lipid groups.

• As stated above, it makes no sense to analyse associations of SNPs with lipid levels without taking account of lipid-lowering treatment.

Re: Thank the reviewer to emphasize this point again. Actually, we took into account of lipid-lowering treatment. In order to prevent lipid-lowering treatment from interfering with the true blood lipid measurements, we required the participants having lipid-treating medicines to suspend the medicine for 12 hours and excluded those who took long-term effect medicines after face to face questionnaire surveys.

6. PLOS authors have the option to publish the peer review history of their article (what does this mean?). If published, this will include your full peer review and any attached files.

Re: Yes, it seems OK for us to publish the peer review history.

If you choose “no”, your identity will remain anonymous but your review may still be made public.

Do you want your identity to be public for this peer review? For information about this choice, including consent withdrawal, please see our Privacy Policy.

Reviewer #1: Yes: Yaxsier de Armas Rodríguez

Reviewer #2: No

[NOTE: If reviewer comments were submitted as an attachment file, they will be attached to this email and accessible via the submission site. Please log into your account, locate the manuscript record, and check for the action link "View Attachments". If this link does not appear, there are no attachment files to be viewed.]

While revising your submission, please upload your figure files to the Preflight Analysis and Conversion Engine (PACE) digital diagnostic tool, https://pacev2.apexcovantage.com/. PACE helps ensure that figures meet PLOS requirements. To use PACE, you must first register as a user. Registration is free. Then, login and navigate to the UPLOAD tab, where you will find detailed instructions on how to use the tool. If you encounter any issues or have any questions when using PACE, please email us at figures@plos.org. Please note that Supporting Information files do not need this step.

Re: We only have figures in a Supporting Information file.

In compliance with data protection regulations, you may request that we remove your personal registration details at any time. (Remove my information/details). Please contact the publication office if you have any questions.
